# The value of social practice theory for implementation science: learning from a theory-based mixed methods process evaluation of a randomised controlled trial

**DOI:** 10.1186/s12874-020-01060-5

**Published:** 2020-07-06

**Authors:** Julia Frost, Jennifer Wingham, Nicky Britten, Colin Greaves, Charles Abraham, Fiona C. Warren, Hasnain Dalal, Rod S. Taylor

**Affiliations:** 1 Institute of Health Research, University of Exeter College of Medicine and Health, St Luke’s Campus, Heavitree Road, Exeter, Devon EX1 2LU UK; 2 Primary Care Research Group, University of Exeter College of Medicine and Health, Exeter, Devon EX1 2LU UK; 3 Institute of Health Research, University of Exeter College of Medicine and Health University of Exeter, St Luke’s Campus, Heavitree Road, Exeter, EX1 2LU UK; 4 Psychology Applied to Health, School of Sport, Exercise and Rehabilitation, University of Birmingham, Birmingham, B15 2TT UK; 5 School of Psychological Sciences, Faculty of Medicine, Dentistry and Health Sciences, School of Psychological Sciences, University of Melbourne, Melbourne, VIC 3010 Australia; 6 Institute of Health Research, University of Exeter College of Medicine and Health, Exeter, EX1 2LU UK; 7 Medical Statistics, Exeter Collaboration for Academic Primary Care, University of Exeter College of Medicine and Health, St Luke’s Campus, Heavitree Road, Exeter, Devon EX1 2LU UK; 8 University of Exeter College of Medicine and Health (Truro Campus), Knowledge Spa, Royal Cornwall Hospital, Truro, TR1 3HD UK; 9 Health Services Research, Institute of Health Research, University of Exeter College of Medicine and Health, St Luke’s Campus, Heavitree Road, Exeter, Devon EX1 2LU UK; 10 Institute of Health and Well Being, University of Glasgow, MRC/CSO Social and Public Health Sciences Unit, University of Glasgow 200, Renfield Street, Glasgow, G2 3AX UK

**Keywords:** Behaviour change, Context, Fidelity, Heart failure, Implementation science, Process evaluation, Randomised controlled trial, Social theory, Tailoring

## Abstract

**Background:**

Although there is trial evidence that complex interventions are effective for the self-management of heart failure, little evidence supports their effectiveness in routine practice. We used Social Practice Theory to guide a Type 1 Hybrid Trial: a mixed methods process evaluation of a complex intervention for heart failure. The objective of this paper is to explore the value of Social Practice Theory for implementation science.

**Methods:**

Social Practice Theory informed a mixed methods process evaluation of a multi-centre randomised controlled trial of a 12 week home-based intervention to optimise self-care support for people with heart failure and their caregivers - Rehabilitation EnAblement in Chronic Heart Failure (REACH-HF). Interviews were conducted with 19 people with heart failure and 17 caregivers at 4 months and 12 months after recruitment into the trial. Cases were constructed at the level of the individual, couple, facilitator and centre; and included multi-modal process and outcome data. Evaluative coding and subsequent within- and cross-case analyses enabled the development of a typology of relationships linking fidelity of intervention delivery and tailoring of content to individual needs and concerns. Social Practice Theory was used to interrogate the relationships between elements of the intervention and their implementation.

**Results:**

Of 216 trial participants, 107 were randomised to the intervention (REACH-HF plus usual care). The intervention was most effective when fidelity was high and delivery was tailored to the individual’s needs, but less effective when both tailoring and fidelity were low. Theory-based analysis enabled us to model complex relationships between intervention elements (competencies, materials and meanings) and social context. The findings illustrate how intervention fidelity and tailoring are contextual and how the effectiveness of the REACH-HF intervention depended on both optimal alignment and implementation of these elements.

**Conclusion:**

The study demonstrates the utility of theory-based analysis which integrates data from multiple sources to highlight contexts and circumstances in which interventions work best. Social Practice Theory provides a framework for guiding and analysing the processes by which a complex intervention is evaluated in a clinical trial, and has the potential to guide context-specific implementation strategies for clinical practice.

**Trial registration:**

ISRCTN, IISRCTN86234930. Registered 13th November 2014.

## Background

Although there is evidence that complex interventions are effective for the self-management of heart failure, many are not implemented in routine practice. Exercise based cardiac rehabilitation in patients with heart failure have shown improvements in health-related quality of life and a reduction in hospitalisations [1]. Whilst evidence for the added value of centre and group-based cardiac rehabilitation for patients with heart failure is strong, uncertainty remains about the effectiveness of alternative modes of delivery [2].

Previous research has failed to describe the complexity of living with heart failure and the burden of self-management and adherence to intervention regimens [3]; failed to identify elements which consistently improve outcomes in self-management interventions for people with heart failure [4]; and failed to explain how programme elements of cardiac rehabilitation interventions interact [5]. Systematic reviews of randomised controlled trials for heart failure have also identified a lack of process evaluations assessing change processes which may inform wider implementation [6].

The Rehabilitation Enablement in Chronic Heart Failure (REACH-HF) multicentre randomised controlled trial demonstrated the clinical effectiveness and cost-effectiveness of the REACH-HF cardiac rehabilitation and self-care intervention for patients with heart failure with reduced ejection fraction (HFrEF) and their caregivers [1, 7, 8]. The randomised controlled trial subsequently demonstrated a statistically significant and clinically meaningful between-group (intervention-control) difference in the Minnesota Living with Heart Failure Questionnaire (MLHFQ) score of − 5.7 points (95% confidence interval − 10.6 to − 0.7) in favour of the REACH-HF intervention group (*p* = 0.025) at 12 months (minimal clinically important difference (MCID) for MLFHQ is 5 points; a higher score indicates a worse clinical outcome) [7].

In this paper we report the results of the theory-based process evaluation and identify the extent to which Social Practice Theory has the potential to progress our understanding from a pragmatic trial of a clinical intervention at T_2_ (translation to patients), to the development of an intervention strategy for implementation at T_3_ (translation to practice) [9, 10], e.g. via the identification of configurations of intervention elements [11, 12].

While the MRC framework for process evaluations of complex interventions remains a valuable tool for exploring mechanisms of impact, debates regarding the role of mechanisms in complex interventions and adaptation to context remain unresolved [13]. Trialists have previously called for greater theorising and understanding of the social aspects of complex interventions [14–16]. Paying attention to social context can improve the effectiveness of interventions in a given context; promote sustainability of an intervention in that context; and promote dissemination in other contexts [17, 18]. More specifically, self-management interventions for chronic conditions could be considerably more effective if the experiences, social and contextual interactions and sense-making processes of both patients and health professionals were prioritised in trial design, evaluation and implementation.

Normalisation Process Theory (NPT) has provided a mid-range theory to guide the implementation and evaluation of complex interventions [19], but the enduring gap between research and implementation suggests that identification of other potential solutions is warranted [20]. While the focus of the NPT framework is on the work that professionals do to ensure that interventions become normalised, less attention has been given to the identification and evaluation of the elements that constitute the mechanisms of impact and how they may or may not operate in a given context [21]. Braithwaite et al. argue that a linear pipeline model of implementation demotes contextual characteristics to ‘confounders’, rather than viewing them as the normal conditions of the practice of healthcare [22]. This perspective is supported by several authors, who have reported on the proliferation of behaviour change interventions that focus mainly on intra-individual processes of change, while identifying that processes related to social context have been overlooked [23, 24].

### Social practice theory

One way to operationalise the evaluation of an intervention ‘in context’ is via Social Practice Theory. This theory has proven popular in environmental research, where it has identified the systemic failure of interventions to change behaviour [25, 26]. In contrast to linear models of implementation, Social Practice Theory offers a more dynamic framework because, rather than focusing on individuals or institutions or programme theories, it is concerned with the implementation of ‘practice bundles’ or sets of interconnected elements and the ways in which practices are reproduced, maintained, stabilised, challenged and surpassed [25, 27].

Social practice is constituted by interdependencies between diverse elements including bodily activities (e.g. exercising), mental activities (e.g. undertaking crosswords for relaxation), material objects (e.g. use of scales for weighing) and their use, background knowledge (e.g. from lay and professional carers) in the form of shared understandings, states of emotion (e.g. anxiety) and motivational knowledge (e.g. the value of maintaining social activities) [11]. Practices have 3 elements: 1) *Materials* including technologies, tangible, physical entities, and the stuff of which objects are made; 2) *Competences* which encompasses skills, knowhow and techniques; and 3) *Meanings* which include symbolic or shared meanings, social norms, ideas and collective aspirations. These elements are the ‘building blocks of practice’, which allow us to describe processes of transformation, diffusion and circulation [11]. For practices to be sustainable they need to capture and retain practitioners who are willing and able to enact these integrating processes and who are therefore willing and able to keep the practices alive. The value of this approach is that it allows us to map the uneven landscapes of opportunity and unequal patterns of access between participants; enabling us to identify the relatively privileged (with the physical, cognitive or socio-economic means to engage), and the marginalised (i.e. the socially excluded who are lacking opportunities to become ‘carriers of practice’) [28, 29].

### Brief description of the REACH-HF trial

The aim of the Rehabilitation Enablement in Chronic Heart Failure (REACH-HF) programme of research was to develop and evaluate a 12-week, home-based facilitated heart failure manual to enhance the quality of life and self-care of people with heart failure and their caregivers (Table 1) [1]. The support needs of people with heart failure and their caregivers were established, and the REACH-HF intervention developed by an intervention mapping process and feasibility study [31].

**Table 1:** REACH-HF components

The REACH-HF intervention consists of four components:	
1	The intervention manual is intended to support people with heart failure and their carers. It contains sections about the condition itself and a range of self-management strategies including medication management, wellbeing and a traffic light algorithm for appropriate help seeking. The manual contains a choice of two structured exercise programmes: a) a chair-based exercise DVD with seven levels of progressively increasing intensity; b) a walking exercise programme aiming to improve the patient’s fitness over time, by encouraging longer walks and a gradually increasing pace, according to each person’s capacity Some of the material from the Heart Manual has been developed as self-help exercises and recorded on CD, to help people relax and manage their breathing [30].
2	The Progress Tracker booklet is intended to encourage patients to monitor their own activities. Regular use of the booklet is intended to help people learn from their own experiences and develop a better understanding of how self-management affects their symptoms and wellbeing.
3	The Family and Friends Resource is a companion manual providing information for carers, in relation to caring for the patient as well as caring for themselves
4	Facilitators: A three day training course for facilitators promoted the use of person-centred counselling, individual assessment and tailoring of the REACH-HF intervention resources to the individual needs of patients and their caregivers.

The REACH-HF randomised controlled trial was conducted at 4 centres in the UK, and a theory-based process evaluation undertaken to assess fidelity of delivery (using a checklist applied to recordings of facilitator-patient-caregiver interactions), and to characterise patients’ and caregivers’ observed and self-reported responses to the intervention [7, 32]. ‘The aim of this paper is to use Social Practice Theory to identify mechanisms through which the intervention did or did not work, and consider the implications for subsequent implementation in routine clinical practice.

### Contributions to the literature

- We undertook a Type 1 Hybrid Trial (a randomised controlled trial evaluating the effect of a complex intervention for the self-management of heart failure, and simultaneously collected longitudinal process data about how the intervention was implemented) and used a rigorous mixed methods approach to explore data from multiple sources.
- Social Practice Theory was used to map and interrogate the relationships between the various elements (materials, competencies and meanings) of the intervention and their implementation in a range of clinical contexts over time.
- Social Practice Theory enabled us to identify that the REACH-HF intervention was optimised when the competencies of the facilitator and the intervention materials were tailored to the goals and needs of the participant in ways which enabled participants to transform their meaning and develop sustainable and flexible self-management practices - but only where the magnitude of any complex problems were within the scope of the intervention.
- Our findings suggest that Social Practice Theory has the potential to progress our understanding from a trial of a clinical intervention at T2 (translation to patients), to guide the development of intervention strategies for implementation at T3 (translation to practice) via the identification of the most appropriate configurations of bundles of intervention elements for implementation of personalised interventions at scale.

## Methods

### Study design

We conducted a theory-based process evaluation of a randomised controlled trial evaluating the effect of the REACH-HF intervention for the self-management of heart failure [1]. We employed a Hybrid Type 1 trial design which both tested the effectiveness of the evidence based intervention and collected data on the implementation processes [12]. The methods of both the REACH-HF randomised controlled trial and process evaluation have been described in detail [7, 32].

Two hundred sixteen patients and 97 caregivers were recruited to the randomised controlled trial from 4 geographical regions across the UK (Birmingham, Cornwall, Gwent and York). One hundred seven patients were randomised to the intervention arm of the trial, and 109 to usual care. The sampling frame for the process evaluation was the intervention arm of the randomised controlled trial, from which 19 patients were sampled for maximum variation in their baseline scores for the primary and secondary outcome measures of the trial: The primary outcome was disease-specific health-related quality of life at 12 months measured using the Minnesota Living with Heart Failure Questionnaire (MLHFQ) [33]. Secondary outcomes were death, hospitalization, generic quality of life (five-dimension EuroQol (EQ-5D-5L) scale) [34], psychological wellbeing (Hospital Anxiety and Depression Scale (HADS)) [35], exercise capacity (incremental shuttle walk test) [36] and physical activity assessed using a GeneActiv accelerometer [37]. Additional secondary measures included the HeartQoL questionnaire [38] and Self-Care of Heart Failure Index [39]. At the baseline clinic visit sociodemographic data and information on past medical history from primary and secondary care records were collected, including New York Heart Association classification [40], key comorbidities, concomitant cardiac drugs and presence of implantable cardiac devices.

### Data collection

Both patients and caregivers were contacted by the interviewer and, with their consent, interviewed 4 months and 12 months after the baseline study enrolment visit. Face to face or telephone interviews were conducted by experienced qualitative researchers (JW, LM) and, with participant consent, audio-recorded and transcribed. A semi-structured interview schedule enabled the interviewers to explore existing self-care practices, perceptions of the interaction with the facilitator and the extent to which different aspects of the intervention were engaged with. As well as assessing behaviour change, we also sought to explore contextual factors which may have facilitated or inhibited change, and the experience of living with progressive chronic heart failure more generally, and change over time.

For the 19 people with heart failure and 17 caregivers who constituted our sampling frame (2–3 patients per facilitator across the 4 regions), all face-to face meetings with the facilitator (typically one 90 min and two to three 45 min), and subsequent telephone contacts (typically 3–6 per patient) were audio-recorded by the facilitator. A 13-domain fidelity checklist was developed and piloted during the feasibility study, and used in the process evaluation to assess the facilitators’ delivery of the intervention. The intervention designers (JW, CG) applied the checklist to the audio recorded intervention sessions [31]. Fidelity scoring attributed a numerical value (0–6) for each of the 13 domains on the fidelity checklist. A purposive sample of audio recordings were used to identify examples of optimal and sub-optimal delivery fidelity (chosen on the basis of maximum diversity of fidelity scores between patients, and where dissonance was identified by JW and CG). Ethical approval was granted by the North West- Lancaster Research Ethics Committee; the trial registration number is ISRCTN86234930.

### Data analysis

Multi-modal data from both the randomised controlled trial and process evaluation were managed with Stata v14.2 [41] and Nvivo 11 Pro [42] and assigned to cases at four levels: all data for a patient/caregiver; dyads of patients and caregivers; all data for each facilitator; and each centre. Multi-modal cases were then created for each patient/caregiver [43], including interview transcripts, contact sheets, audio recordings of facilitator-patient sessions and associated fidelity scores, summaries of interactions, field notes, clinical data such as case-report forms, and patient reported outcome measures.

Interview transcripts were subject to a preliminary cycle of evaluative coding, which assigned a judgement about the value or significance of the intervention [44]. This noted the presence or absence of an element (e.g. chair based or walking exercise) and the extent to which it was positively or negatively evaluated by the patient/caregiver. This amplified the fidelity scores, which attributed a numerical value (0–6) to the facilitation as outlined in the facilitator training manual. A secondary cycle of explanatory coding extended the mapping of variance across the dataset, and enabled within-case analyses over time, cross-case analyses of intervention elements and social and contextual variables [44], and the further identification of directional processes [45]. *Fidelity* was coded on the basis of the emphasis given to the intended core components of the intervention, while *tailoring* was coded in relation to the spirit of the intervention being adapted to the local context in order to achieve a ‘good ecological fit’ [13].

Social Practice Theory was used to further interrogate the relationships between the various elements of the intervention [11]. While JF coded the data, the iterative analysis was undertaken through extensive team discussions with JW who had collected most of the data, produced extensive field notes, reviewed the transcripts and assessed the audio-recorded facilitation sessions for fidelity, and NB who independently annotated the content of purposively sampled facilitated sessions. Crucially, the qualitative data collection and preliminary analysis was completed before the overall trial outcome data were known, to minimise bias in interpretation [16, 43]. Analysing the longitudinal data from all 19 cases over two time-points [43, 45] enabled the development of a typology of relationships exploring fidelity of intervention delivery and tailoring of content to individual needs and concerns..

## Results

Repeat semi-structured interviews were conducted with 19 patients (14 at 12 months) and 17 caregivers (15 at 12 months) between August 2015 and August 2016. Baseline demographic and health characteristics of the 107 REACH-HF patient participants in the intervention arm are set out in Table 2, with characteristics of the 19 interviewed participants shown for descriptive comparison. Data has been anonymised.

**Table 2:** Baseline characteristics of patients with Heart Failure participating in the Process Evaluation

Characteristic	REACH-HF ***n*** = 107 (%)	Qualitative interview sample ***n*** = 19 (%)
**Age (years), mean (SD)**	69.7 (10.9)	68.5 (9.8)
**Female sex**	26 (24)	7 (37)
**BMI (kg/m**^**2**^**); mean (SD)**	29.5 (6.6)	31.5 (7.4)
**Main activity**		
Retired	81 (76)	15 (79)
In employment or self-employment	18 (17)	2 (11)
Unemployed	5 (5)	1 (5)
Other	3 (3)	1 (5)
**Ethnic origin**		
White	100 (93)	19 (100)
Other (Black, Asian, other)	7 (7)	0 (0)
**NYHA status**		
Class I	24 (22)	0 (0)
Class II	63 (59)	13 (68)
Class III	20 (19)	6 (32)
**Ischaemic aetiology of HF**	48 (45)	9 (47)
**Time since diagnosis of HF (years)**		
< 1	35 (33)	6 (32)
1–2	18 (17)	1 (5)
> 2	54 (51)	12 (63)
**LVEF (%); mean (SD), n**	32 (8), 76	27 (9), 11
**NT-pro-BNP level (pg/mL); mean (SD)**	1460 (1928)	1321 (1123)
**Current smoker**	6 (6)	1 (5)
**Comorbidities (past or present)**		
Diabetes mellitus	26 (24)	5 (26)
Myocardial infarction	29 (27)	7 (37)
Hypertension	45 (42)	9 (47)
Chronic renal impairment	14 (13)	3 (16)
Arthritis (osteoarthritis or rheumatoid)	45 (42)	11 (58)
Atrial fibrillation or atrial flutter	48 (45)	9 (47)
COPD	9 (8)	2 (11)
Depression	27 (25)	7 (37)
**Number of comorbidities**		
0	63 (59)	9 (47)
1	32 (30)	7 (37)
2	7 (7)	1 (5)
3	5 (5)	2 (11)
**Baseline use of drugs**		
Beta-blocker	90 (84)	18 (5)
Angiotensin II receptor antagonist	31 (29)	8 (42)
ACE inhibitor	68 (64)	10 (53)
**Baseline use of devices**		
ICD	10 (9)	3 (16)
CRT	10 (9)	1 (5)
Combined CRT/ICD	5 (5)	2 (11)
Pacemaker	11 (10)	1 (5)
**Location**		
Cornwall	30 (28)	7 (37)
Gwent	23 (22)	3 (16)
Birmingham	27 (25)	4 (21)
York	27 (25)	5 (26)
**Caregiver present at randomisation**	53 (50)	13 (68)

Our in-depth analysis of this multi-modal data enabled us to create a 2 × 2 typology of fidelity and tailoring [46]. Here we provide four in depth illustrative cases (Table 3) that represent each of the possible combinations of fidelity and tailoring, and which illuminate the strengths and weaknesses of the REACH-HF intervention when viewed from a social practice perspective. These cases were selected for comparative purposes and each typifies one quadrant of the table, and demonstrate the results of our theory-based analysis (Table 4). High fidelity and good tailoring enabled facilitators to improve patients’ competencies, develop appropriate meanings and provide suitable materials. Conversely, poor fidelity and/or poor tailoring meant that some or all of patients’ competencies, meanings or materials were not enhanced, particularly in situations of little social and material capital. Not all cases were clear cut.

**Table 3:** Illustrative cases: Baseline Data

	Good tailoring	Poor tailoring
**Good fidelity**	**Mary** 74 year old female Retired, non-smoker HF for 3 years Comorbid breast cancer, arthritis, chronic back pain, vaginal prolapse Medications: A2 receptor agonist, beta-blocker, loop diureticNYHA Class II (Mild) Ejection fraction Severe (< 35%) HADS Anxiety (1) Depression (2) MLHFQ (50) Lives with husband in redeveloped semi-detached council house. Cared for by her 3 daughters.	**John** 71 year old male Retired, smoker HF for 6 months Comorbid chronic back pain and depression. Medications: ACE Inhibitor, Aldosterone receptor agonist, beta-blocker, loop diureticNYHA Class II (Mild) Ejection fraction Severe (< 35%) HADS Anxiety (7) Depression (9) MLHFQ (50) Lives alone (divorced) in privately rented first floor flat, waiting to be rehoused in social housing. Daughter visits, but not designated carer.
**Poor fidelity**	**Helen** 68 year old female Retired, ex-smoker HF for 7 months Comorbid lung cancer, arthritis, back pain depression Medications: A2 receptor agonist, beta-blocker, loop diuretic NYHA Class III (Moderate) Ejection fraction Severe (< 35%) HADS Anxiety (19) Depression (11) MLHFQ (Missing) Lives with husband (also her carer) in privately owned bungalow, built by her husband	**Dorothy** 73 year old female Retired, non-smoker HF for 6 months No comorbid conditions Medications: ACE Inhibitor, Aldosterone receptor agonist, beta-blocker, loop diuretic NYHA Class II (Mild) Ejection fraction Severe (< 35%) HADS Anxiety (4) Depression (2) MLHFQ (Missing) Lives alone (widowed) in privately owned semi-detached house. Cares for her grandchildren.

These four illustrative cases demonstrate the interplay between materials, meanings and competencies. Names are pseudonyms

**Table 4:** Typology of Fidelity and Tailoring: Longitudinal data

	Good tailoring	Poor tailoring
**Good fidelity**	**Mary** Facilitator Baseline notes: “Realises that she is not exercising much but is active all day. Would like to walk but is limited by [vaginal] prolapse, limited by backache (osteopenia) and osteoarthritis … Occasional walk around shopping centre or park with daughter. Nervous to go alone. (daughters) very supportive and keen to understand and help” (Facilitator 1, initial contact sheet) Facilitator baseline assessment: Facilitator: Is there anything else at the moment that you want to talk about to do with your heart condition? Mary: when they say like with the heart is weak or heart failure part I mean, that, what is, that means the heart is *weaker*? Facilitator: yes the heart muscle it has been damaged…or become stretched you know and isn’t, when the muscles *contract*, whether it’s your arm muscle or your leg muscle or your heart muscle, it sort of contracts and squeezes…yes that’s right and it’s not squeezing quite as much as it should do, so not quite so much blood is actually getting circulated each time. So the reason why you get breathless is because your heart is not pumping enough blood and oxygen around the body. So your breathing is a sort of automatic reaction for it…. (Facilitator 1 Initial assessment) Patient interview at 4 months: “when [facilitator] was there she never rushed us, we’d go through things steadily page by page on the book I was writing on…Progress Tracker....she spent the time and she explained things and I found it very good. She also just let me chat about what benefits I was finding from it” (Mary 4 month interview) Patient interview at 12 months: “the knowledge I gained from [facilitator], the information, the questions that I asked right at the beginning…were probably some of the most important ones….she was able to not just answer it but explain it as well…I’m a person that is, if you want to tell me something, tell me so that I can understand it…but if it’s also written down, I can also get more knowledge…often more than just talking about it, ‘cos you take it in just a bit at a time” (Mary 12 month interview) Facilitator mid-intervention contact sheet: “Phoned to check on progress. However she was in bed with a very bad [urinary tract infection]. Seen by GP and on antibiotics. Told to drink plenty of water. She was unsure what to do as limited to 1 l [due to severity of HF]. Advised that the GP would know that she is restricted yet has advised the extra drinks to flush thro’ the infection. If she’s worried she should contact the GP or [HFCNS]…” (Facilitator 1, subsequent contact sheet) Patient interview at 4 months: “I have sometimes to draw my breath in because they [my daughters] smother me…the surgeon that put the stent in my heart… he told my daughters and my husband:” “I’ve only done one part, the rest of it is medication to help support your heart. Your heart is very, very weak… I never clocked that they were so worried” (Mary 4 month interview) Patient interview at 12 months: “I’m very pleased with how I’ve got on since I started this [programme]… as I was looking at life before then, that this is how I was going to be, spending most of my time in the chair, and looking at the place going:” “Oh I wish I could do this, I wish that I could do that, and now I don’t wish…there’s a total difference to me to what there was” (Mary 12 month interview)	**John** Facilitator Baseline notes: “First home visit – introduction to the manual- Discussed patient’s understanding of HF manual and information given as to the purpose of my visits – History taken from patient – social support discussed. Issues around housing discussed…signposted to the manual” (Facilitator 2, initial contact sheet) Facilitator final notes: “Home circumstances big concern for [John], listened to [John]‘s concerns with landlord/electric/gas/rehousing. Awaiting rehousing [John] has all the relevant contact numbers... Discussed how I felt [John] had benefited from manual – better understanding of heart failure, medications, signs and symptoms etc.” (Facilitator 2, final contact sheet) Patient interview at 4 months: “I don’t think it put a lot of my mood in [Progress Tracker] I’d’ve put good every time...the biggest problem I’ve had is with the gas board. I did put shopping, hobbies as well...The biggest difference is when I’m going to move into this bungalow, I mean I’m a smoker, I have cut down a hell of a lot...It’s a no smoking area” [John, 4 month interview]
**Poor fidelity**	**Helen** Facilitator initial contact: “How she was – emotional. Very anxious about condition. Scared she might die suddenly...Explored support from [local agency] and areas of manual to try techniques to relieve stressful symptoms...” (Facilitator 3, initial contact sheet) Patient interview at 4 months: “I was really getting very depressed...I’m just heaps better than before...And if it wasn’t for being on the [REACH-HF] study, I don’t think I’d be walking like I am now.” (Helen, 4 month interview) “Interviewer: Did you show the manual to anybody else?Helen: No, I hid it in a drawer. I didn’t want the children to see it...and then I got to the CPR bit...I didn’t want the children to know that something could happen to me suddenly... when they were gone I’d take it out, because I had to remember to write my daily strides in it.” (Helen, 4 month interview) Patient interview at 12 months: “Interviewer: Have you gone back [to the manual]? Helen: No, I’m sorry, every time I pick up the manual I see the terminal bit of it, so I haven’t... when I read things like that...I tend to add a little bit, or read things into it. My brain goes: zoom, to the point of no return.” (Helen, 12 month interview) By failing to increase her knowledge and understanding about HF, Helen learned by rote and was very proud of recording her 1900 steps a day in the Progress Tracker, which reflected the focus of the facilitated sessions: Patient interview at 4 months: “[Facilitator] said: it’ll build your heart muscle up... I wasn’t thinking that way, I was thinking: If I rest my heart and don’t do anything...keep me going a bit longer... And the more I thought about walking, and my heart muscle and all, the more, well, I thought: Well, I’ll walk and try and get stronger” (Helen, 4 month interview) Facilitator final contact sheet: “How’s she feeling – good about her heart. Worried about lung. Good days but more bad days. Previously not answering phone. Feeling a little brighter now...Weight decreased. Not eating. Discussed nutritional drinks from GP. Doesn’t want to contact GP. Not renewed prescription for Valium...Me to contact GP re: nutritional drinks and Valium” (Facilitator 3, final contact sheet)	**Dorothy** Facilitator initial contact sheet: “Introduction to REACH, myself and manuals. Discussion surrounding use of Progress Tracker, traffic light system and signs and symptoms....” (Facilitator 4, initial contact sheet) Facilitator initial assessment: Facilitator: and what did [Doctor] say about this scan? Dorothy: he was (sighs) do you know what, I do find it confusing. He said something about 40% F: OK D: is that 40% *not* working or 40% working? F: yes, OK D: I’m not sure which way that went F: Ok yeah, I’m not sure about whether it *is* 40% but if … my suspicions are if you say 40%, is that *yes* that it’s working (pause) at 40% of what’s expected, if that makes sense. (Facilitator 4 Initial assessment) Patient interview at 4 months: “Interviewer: Did you try the chair based exercises?” Dorothy: I didn’t, not really. No I didn’t... Sorry [laughter]....I didn’t find it comfortable... I didn’t tend to wear the [pedometer] and when it fell off, I didn’t seem to carry on with it... Interviewer: Are you sort of writing things down anywhere, or monitoring? Dorothy: Oh, now I’ve got to be honest: No I haven’t done, no....the manual tend to just flick through and just read different pages you land on … “Interviewer: Was there anything that you didn’t like [about the facilitator], I’m sensing that you’re a little bit reserved? Dorothy: Didn’t seem reliable...I just felt it wasn’t constant.” (Dorothy 4 month interview). Patient interview at 12 months: “I still can’t see myself as a priority...I can’t seem to get the seriousness of it...I seemed to think that because I was busy, I’m exercising but it’s not, it’s not the same thing really is it? I haven’t kept up [with the monitoring]...I did have a diary at first...No, have got a bit complacent...I don’t know what to look for really... my condition hasn’t changed...” (Dorothy 12 month interview)

### Good fidelity and good tailoring

Mary is a 74 year old woman, diagnosed with heart failure 3 years before the study, who lives with her husband but is cared for by her three daughters. Mary received the lowest ‘dose’ of REACH-HF in these examples (293 min, over three face-to-face and three telephone intervention sessions). At the initial assessment, the facilitator explored Mary’s current understanding of her situation and baseline ‘capacity’ before gradually introducing the REACH-HF manual. This is reflected in the high fidelity scores at the initial intervention session (5 or more in 7 of the 13 domains) and subsequently (4–5 in most domains). At the initial assessment, the facilitator linked the discussion of Mary’s arthritis to her capacity for walking before going on to talk about the exercise component of the manual (e.g. the participant’s existing meaning and competence, and trial materials). This assessment was used to elicit Mary’s highest-priority self-care goal (walking) and the work required to fulfil this. The facilitator provided answers to Mary’s questions (thus improving her competence), as well as providing explicit instances of information to augment Mary’s understanding of her condition. The information provided by the facilitator was reinforced with the tailored use of the manual, which enabled Mary to create new understanding and meaning around her heart failure.

At 12 months Mary was clear that her enduring use of the manual after the intervention period improved her self-care. To sustain change over time, the facilitator drew on Mary’s existing relationships with her heart failure specialist nurse (HFSN) and General Practitioner (GP), and signposted her to their continued support. As well as drawing on the competencies of other professionals, this skilled facilitator was also able to transform family support, in response to Mary’s suggestion that she was feeling stifled by her daughters. Subsequently Mary’s daughters worked with the facilitator and the Friends and Family resource to support Mary in reaching her goals. Mary’s daughter annotated the Progress Tracker with stickers to help navigate key tasks, as well as developing a one-page tailored ‘tracker’ for Mary to write down her exercise, signs and symptoms, relaxation activities and sleep pattern after the intervention period. This enabled Mary to continue learning and develop her new found autonomy as her walking and confidence improved.

By using the initial assessment session to take a comprehensive medical history and explore Mary’s goals, this facilitator was able to use her time most efficiently and tailor the intervention to Mary’s needs. The facilitator also engaged her social support network to involve a series of others in working with Mary to achieve her goal and meet her needs. By working with Mary’s existing social resources (both lay and professional) this facilitator enabled self-care practices (e.g., walking) and social support systems to develop, which changed the meaning that Mary attributed to living with heart failure and enhanced her coping competencies.

### Good fidelity and poor tailoring

While the intervention was tailored to Mary’s needs with good effect, John’s enduring socio-economic circumstances inhibited the effectiveness of the intervention. John is a 71 year old divorced man with depression, who smokes cigarettes and was diagnosed with heart failure 6 months previously. Living alone in a privately rented first-floor flat, but about to move into ground-floor social housing, he was troubled by large debts to utility companies. Despite suggesting that he had lived with heart failure since having rheumatic fever at 8 years old, John demonstrated that he did not understand how his heart worked to support activity.

Although the facilitator took a medical history and introduced the elements of the intervention, John spent much time talking about his stressful financial situation. Running out of time, the facilitator was unable to get beyond John’s social problems and did not elicit a self-care goal from John to underpin his rehabilitation. Thereafter the conversation remained facilitator-led, with little evidence of rapport building. Instead the overarching concern remained debt and over time tailoring was diminished as the facilitator adopted a more didactic style. Towards the end of this initial assessment session, the facilitator drew on her professional repertoire and provided John with a plumbing metaphor to explain how his cardiovascular system works, but while she proceeded with her explanation John said “you’re rattling on now” and she did not tailor the session to his response. This is reflected in the facilitator notes for this session which detailed her interpretation of the session rather than John’s.

Throughout the intervention period John continued to record his mood as ‘good’ in the Progress Tracker despite his enduring problems, and he provided minimal details about his exercise and medication use, other than noting that he gained weight. John subsequently withdrew from the randomised controlled trial and process evaluation, but the focus when interviewed at 4 months was about a perceived better future, rather than present, and his motivation for cutting down smoking was due to planned accommodation changes, rather than any new meaning attributed to REACH-HF.

While Mary’s facilitator engaged Mary’s existing support network to supplement her behaviour change, John has little social or material capital to draw on. His underlying depression and perception of his current circumstances are such that he is unable to engage with the new material and competencies that REACH-HF provide in a way that would enable him to construct a new meaning or self-care practices around his heart failure. Although the facilitator fully acknowledged his distressing circumstances neither her existing competencies nor the intervention materials could be tailored to mitigate such extensive socio-economic deprivation.

### Poor fidelity and good tailoring

Sixty-eight year old Helen received one of the highest doses of REACH- HF at 470 min, over 4 face-to-face and 7 telephone sessions with her facilitator. She was recently diagnosed with heart failure and a lung ‘nodule’, and has long standing anxiety and depression, with some of the highest HADS scores (Anxiety = 19, Depression = 11) in the wider trial population at baseline. Helen lives with her husband who is her caregiver, but who struggles to deal with her emotional state. Unlike John, Helen had many interactions with multiple health professionals in the 6 months prior to the trial. Helen’s facilitator began by dealing with Helen’s concerns head-on: unpacking her fear of dying. This is one of the few cases in which the facilitator demonstrated the required exercise level to the patient, by actively walking with her and demonstrating warming up, cooling down, and the required effort for exercising the heart, which Helen valued.

However, while Helen was happy to do the physical exercise that the facilitator suggested, she did not fully engage with the heart failure manual – hiding it from her family and ignoring the sections which would have increased her own learning about the severity of her condition. By failing to increase her knowledge and understanding about heart failure, Helen learned by rote and was very proud of recording her 1900 steps a day in the Progress Tracker, which reflected the focus of the facilitated sessions.

While improving her exercise capacity was beneficial, this was in the absence of wider learning about her condition which meant that over time 1900 steps became the end rather than the means of improving her quality of life. The facilitator was viewed as kind and caring, but this was to the detriment of challenging Helen’s fears or supporting her autonomy. The subsequent sessions were increasingly didactic, as the facilitator failed to elicit the required response from Helen and instead made assumptions on her behalf. For example in the second audio-recorded session the facilitator asked Helen what her priorities were, but failed to wait for Helen’s reply. Subsequently other missed opportunities for engagement included not reviewing Helen’s physical symptoms and only using the heart failure manual in a responsive way, so that relevant sections were repeatedly omitted. In the final audio-recorded session the facilitator did not check Helen’s understanding of heart failure or explore why she had not contacted a local agency; rather the facilitator took on the task of arranging appointments and medication.

Helen achieved a limited transformation as a result of her involvement in REACH-HF. While the facilitator demonstrated a degree of competence in modelling the required exercise, Helen assumed that the circuit that they covered was sufficient and there was limited use of additional REACH-HF materials. Helen learned that her heart needed to be exercised, but this was achieved via habitual repetition rather than any increased competence or new meaning that would have allowed Helen to tailor her exercise to the weather or extend it to improve her mental health. Furthermore, many of her existing behaviours around denial and lack of agency went unchallenged.

### Poor fidelity and poor tailoring

The final case is Dorothy, a 73 year old widow who lives alone and often looks after her grandchildren. She was diagnosed with heart disease 6 months before the trial, and while struggling to come to terms with her diagnosis was one of the sickest patients in the process evaluation, with an ejection fraction of only 21% at baseline cardiac assessment.

This example is striking for what was absent. The audio-recording of the initial session began with the introduction of the REACH-HF manual, and there was no discussion of Dorothy’s medical history, goals, or social situation. Here, fidelity scores were low at baseline and subsequently (all under 2.5 and deemed inadequate). The facilitator neither elicited or addressed Dorothy’s concerns despite her being newly diagnosed, having seen no health professionals since diagnosis, and having a limited understanding of her condition.

This also seems to be an example of lack of professional competence, as the facilitator was unable to answer adequately a straightforward question about Dorothy’s heart failure. While Dorothy had little understanding of her heart failure, the facilitator lacked the competence to improve her learning, as demonstrated in a subsequent session when Dorothy asked a question about medication and the facilitator neither found the relevant section in the manual nor drew upon any wider professional knowledge. Because the facilitator continually failed to engage Dorothy in the programme, Dorothy gained little understanding of the utility of the trial materials, and suggested that she had little faith in the facilitator’s competence. Post- intervention Dorothy was still struggling to reconcile that she had a chronic condition that requires self-care. She admitted that she would let her weight increase by seven to fourteen pounds (potentially fatal) before she would call for professional help.

Dorothy lacked the skills and competence to care for her heart failure, and having a facilitator who failed to challenge her existing interpretations ensured that Dorothy did not engage with the REACH- HF intervention. Like Helen, Dorothy now has a walk that she undertakes regularly, but she has been unable to develop any meaningful notion of her heart failure, as she still attributes her lack of deterioration to the lack of severity of her heart failure, whereas paradoxically any subsequent deterioration would be attributed to “just my age”.

## Discussion

### Summary of key findings

This is the first process evaluation of an evidence-based home-based rehabilitation intervention to optimise self-care support for heart failure patients and their caregivers. We identified that the intervention was most effective when fidelity was high and delivery was tailored to the individual’s needs, and that the intervention was less effective when both tailoring and fidelity were low. Furthermore, our theory-based analysis enabled us to model complex relationships between intervention elements (competencies, materials and meanings) and social context. For example, in our ‘best case’ the meaning that Mary attributed to living with heart failure was transformed by the strategic use of professional competencies (skilled goal elicitation), purposeful use of materials (the tailored use of the manual and progress tracker), and the augmentation of meanings (through the continued engagement and support of Mary and her family). Whereas, in our ‘worst case’ Dorothy’s meaning was not transformed because neither the competencies of the facilitator (both heart failure specific and wider professional knowledge) or materials (which were not tailored to needs) were able to engage her in the programme. By using Social Practice Theory to interrogate these relationships between the various elements of the intervention over time we were able to identify how fidelity and tailoring are contextual and how effectiveness of the REACH-HF intervention was dependent on both optimal alignment and implementation of elements. Therefore Social Practice Theory not only progressed our understanding of both the findings and processes of a trial of a clinical intervention but also provided insights to inform the development of strategies for implementation in routine practice [20, 47].

### Interpretation and implications of results

The constituents of the REACH-HF intervention were chosen in response to the challenges of implementing a rehabilitation intervention within the patient’s home [48], based on empirical evidence of their effectiveness, and engagement with stakeholders [31]. While theoretically informed qualitative analyses have previously identified the importance of self-care ‘assemblages’ in routine practice [49, 50], we sought to explore the dynamic interactions of fidelity and tailoring during the delivery of the REACH-HF intervention under trial conditions and subsequently to inform implementation strategies.

By utilising a theory-based mixed methods approach, we were able to identify key practices (such as the health professional knowing when to employ appropriate sections of the manual or implement practices from their wider professional repertoire) as well as configurations of best practice (such as the facilitator demonstrating the required exercise level and tailoring explanations). These demonstrate that persistence and change are dependent upon feedback between one instance or moment of enactment and another, and on patterns of mutual influence between co-existing practices [11].

Our findings indicate that the opportunities for achieving sustained behaviour change are contingent upon what the bundles of intervention practices demand of a person with heart failure and/or their caregiver and on previous life histories and resources accumulated along the way. This suggest that the provision of a simple blueprint or template for implementation into routine practice would be inappropriate considering the potential importance of tailoring the intervention to context and any change over time. Rather than a generalisable prescription our mixed methods approach provides critical transferable insights and greater explanatory power, allowing policy makers and practitioners (both lay and professional) to make informed decisions about future uptake of individual elements, bundles of elements, and the total REACH-HF package more generally [15, 43, 51, 52].

Our findings highlight the importance of one-to-one contact for participants. Thus, although such contact may be expensive, it may be critical to allow flexibility to ensure intervention impact across participants. Our findings also highlight how intervention delivery can vary across personal contexts, so emphasising the need to identify and train facilitators for best practice in intervention delivery before implementation. Our findings emphasise the value of both a Hybrid Type 1 trial design - synthesising randomised controlled trial and process evaluation data - prior to implementation. The integration of process and outcome data over time enabled us to make within-case and cross-case analyses. Such comparisons can highlight how well an intervention is designed to respond to a range of participant needs. This may direct intervention improvement prior to implementation (e.g. more focused facilitator training), as well as future implementation strategies (e.g. that align assessment of contextual factors, identification of patient and clinical goals, and the most appropriate intervention components).

### Implications for research

This paper has a number of implications for future research. Commentators note that theoretical endeavours for implementation science often lack “how-to” support for carrying out implementation evaluation and interpretation [53]; can overlook the extent to which practitioners determine the processes and outcomes of implementation [54]; and the extent to which context is wider than the physical setting in which the implementation takes place [16, 17].

Social Practice Theory enabled us to identify that this complex behaviour change intervention was optimised when the competencies of the facilitator (e.g. rigorous assessment and an ability to challenge lay interpretations) and the intervention materials (e.g. the section of a manual or use of a patient tracker) were tailored to the goals and needs of the participant in ways which enabled them to transform the meaning (e.g. foster an appreciation of the value of exercise), thus enabling them to develop sustainable and flexible self-management practices. Sometimes, this was achieved despite a participant’s enduring problems; but not all facilitators were well acquainted with the intervention, or knowledgeable enough to deliver it well. If the problem is within the scope of the intervention (e.g. anxiety) then the tailoring is appropriate although fidelity may be compromised. However, if the problem is not within the scope of the intervention (e.g. material deprivation), then tailoring is very difficult. Our attention must now turn to how to deliberately steer or target practices and/or bundles of practices in the face of enduring health care and structural inequalities.

Hawe et al. contend that perhaps one of the reasons for interventions not working is that their components have been overly standardised, proposing that thinking about the real world context should become the ideal instead [14]. Our findings suggest that Social Practice Theory provides a framework for us to bring the social context back into process evaluations of complex interventions to inform implementation strategies in a meaningful way [14, 23].

We agree with Blue et al. [50] that complex interventions need to move beyond seeing people as static individual selves that require changing, to engaging people entwined in dynamic social arrangements who are defined and constituted through the practices that they adopt and enact [23]. We suggest that our use of Social Practice Theory to inform our analysis of multi-modal process and outcome data is a robust and dynamic theoretically informed methodological approach for evaluating intervention and implementation practices. We see this as having the potential to illuminate which configurations of elements need to be customised for every ‘user’ - such that any standardisation concerns the *function* of elements or bundles of elements that constitute interventions in a given context (e.g. unpacking Helen’s anxieties about death to enable her to participate in exercise), rather than the current focus on standardising the *form* of elements and their delivery (e.g. the deployment of the manual in Dorothy’s case without wider contextualisation) [55]. We suggest that this can help us moved toward the implementation of personalised interventions at scale [29].

### Strengths and limitations

We have conducted the first mixed methods process evaluations of a comprehensive self-management intervention for people with heart failure and their carers. The use of a well specified sociological theory (Social Practice Theory) enabled the core elements of this complex intervention and their assemblages to be better articulated, explored and explained. This has enabled us to amplify the quantitative trial results and to use these insights to develop our subsequent implementation strategy.

We have acknowledged the limitations of this study elsewhere, in relation to other data we might have collected [32]. Our ongoing research and implementation activities aim to redress some of these limitations and build on our findings, with particular reference to the training and support of the facilitators [56, 57]. We have also noted the lack of diversity of our patient population, and it is also vital to address the particular challenges facing people from black and minority ethnic communities who are not well served by current interventions.

## Conclusion

We conclude that Social Practice Theory has the potential to progress our understanding from a trial of a clinical intervention at T2 (translation to patients), to guide the development of an intervention strategy for implementation at T3 (translation to practice) via the identification of the most appropriate configurations of bundles of intervention elements for implementation in individual real-world clinical scenarios.

## Data Availability

Transcripts will not be shared in their entirety to protect the anonymity of participants and the facilitators delivering the intervention. However, requests for excerpts of the data will be considered on an individual basis. Please contact the corresponding author.

## Abbreviations

ACE: Angiotensin-converting-enzyme inhibitor
BMI: Body Mass Index
COPD: Chronic Obstructive Pulmonary Disease
CRT: Cardiac resynchronisation therapy
HADS: Hospital Anxiety and Depression Scale
ICD: Implantable cardioverter defibrillator
LVEF: Left ventricular ejection fraction
NYHA: New York Heart Association (Classification)
MRC: Medical Research Council
MLHFQ: Minnesota Living with Heart Failure Questionnaire
NPT: Normalisation Process Theory
REACH-HF: Rehabilitation Enablement in Chronic Heart Failure
T2/3: Time 2/Time 3

## References

[1] Taylor RS, Hayward C, Eyre V (2015). The clinical effectiveness and cost-effectiveness of the rehabilitation Enablement in chronic heart failure (REACH-HF) self-care rehabilitation intervention in heart failure patients and caregivers: rationale and protocol for a multicentre randomised controlled trial. BMJ Open.

[2] NICE. Chronic heart failure: management of chronic heart failure in adults in primary and secondary care: NICE ed. Chronic Heart Failure. National Institute for Health and Care Excellence. 2010.

[3] Greenhalgh T, A’Court C, Shaw S (2017). Understanding heart failure; explaining telehealth – a hermeneutic systematic review. BMC Cardiovasc Dis.

[4] Jonkman N, Westland H, Groenwold R, Ågren S, Anguita M, Blue L, Bruggink-André de la Porte P, Dewalt D, Hebert P, Heisler M, Jaarsma T, Kempen G, Leventhal M, Lok D, Mårtensson J, Muñiz J, Otsu H, Peters-Klimm F, Rich M, Riegel B, Strömberg A, Tsuyuki R, Trappenburg J, Schuurmans M, Hoes A (2016). What are effective program characteristics of self-management interventions in patients with heart failure? An individual patient data meta-analysis. J Card Fail.

[5] Clark A (2013). What are the components of complex interventions in healthcare? Theorizing approaches to parts, powers and the whole intervention. Social Sci Med.

[6] Albano M, Jourdain P, de Andrade V, Domenke A, Desnos M, d’Ivernois JF (2014). Therapeutic patient education in heart failure: do studies provide sufficient information about the educational programme?. Arch Cardiovasc Dis.

[7] Dalal H, Taylor R, Jolly K, Davis R, Doherty P, Miles J, van Lingen R, Warren C, Green C, Wingham J, Greaves C, Sadler S, Hilsdon M, Abraham C, Britten N, Frost J, Singh S, Hayward C, Eyre V, Paul K, Lang C, Smith K, on behalf of the REACH-HF Investigators (2019). The effects and costs of home-based rehabilitation for heart failure with reduced ejection fraction: The REACH-HF multicentre randomized controlled trial. Eur J Prev Cardiol.

[8] Taylor RS, Wingham J, Frost J, Britten N, Greaves C, Abraham C, Warren F, Jolly K, Miles J, Paul K, Doherty P, Singh S, Davies R, Green C, Eyres V, Hayward C, van Lingen R, Smith K, Lang C, Hillsdon M, Dalal H. On behalf of the REACH-HF research group. Facilitated self-care and rehabilitation for patients with reduced ejection fraction heart failure (REACH-HF trial): impact on caregiver outcomes. Submitted to European Journal of Cardiovascular Nursing.

[9] Westfall J, Mold J (2007). Fagan L (2007) practice-based research: “Blue highways” on the NIHR roadmap. J Am Med Assoc.

[10] Dougherty D, Conway PH (2008). The “3T’s” road map to transform US health care: the “how” of high-quality care. J Am Med Assoc.

[11] Shove E, Pantzar M, Watson M (2012). The dynamics of social practice: everyday life and how it changes.

[12] Bauer M, Damschroder L, Hagedorn H, Smith J, Kilbourne A (2015). An introduction to implementation science for the non-specialist. BMC Psychology.

[13] Moore G, Audrey S, Barker M, Bond L, Bonell C, Hardeman W, Moore L, O’Cathain A, Tinati T, Wight D, Baird J (2014). Process evaluation of complex interventions: Medical Research Council guidance.

[14] Hawe P, Shiell A, Riley T (2004). Complex interventions: how “out of control” can a randaomised controlled trail be?. Brit Med J.

[15] Oakley A, Syrange V, Bonell C, Allen E, Stephenson J, RIPPLE study team (2006). Process evaluation in randomised controlled trials of complex interventions. Brit Med J.

[16] Hoddinot P, Britten J, Pill R (2010). Why do interventions work in some places and not others: A breast feeding support group trial. Social Sci Med.

[17] Damschroder L, Aron D, Keith R, Kirsh S, Alexander J, Lowery J (2009). Fostering implementation of health services research findings into practice: a consolidated framework for advancing implementation science. Implement Sci.

[18] Grimshaw J, Presseau J, Tetroe J, Eccles M, Franci J, Godin G, Graham D, Hux J, Johnston M, Legare F, Lemyre L, Robinson N, Zwarenstein M (2014). Looking inside the black box: results of a theory-based process evaluation exploring the results of a randomized controlled trial of printed educational messages to increase primary care physicians’ diabetic retinopathy referrals. Implement Sci.

[19] May CR, Cummings A, Girling M, Bracher M, Mair FS, May CM, Murray E, Myall M, Rapley T, Finch T. Using normalization process theory in feasibility studies and process evaluations of complex healthcare interventions: a systematic review. Implement Sci. 2018;13(80). 10.1186/s13012-018-0758-1 PMC5992634 29879986

[20] Whitty C (2015). What makes an academic paper useful for health policy?. BMC Med.

[21] Clarke DJ, Godfrey M, Hawkins R, Sadler E, Harding G, Forster A, McKevitt C, Dickerson J, Farrin A (2013). Implementing a training intervention to support caregivers after stroke: a process evaluation examining the initiation and embedding of programme change. Implement Sci.

[22] Braithwaite J, Churruca K, Ellis L, Herkles J. When complexity science meets implementation science: atheortical and empirical analysis of systems change. BMC Med. 2018;16(63). 10.1186/s12916-018-1057-z PMC5925847 29706132

[23] Craig P, Di Ruggiero E, Frohlich KL, Mykhalovskiy E, White M, On behalf of the Canadian Institutes of Health Research (CIHR)–National Institute for Health Research (NIHR) context guidance authors group (2018). Taking account of context in population health intervention research: guidance for producers, users and funders of research.

[24] Holman D, Lynch R, Reeves A (2018). How do health behaviour interventions take account of social context? A Literature trend and co-citation analysis. Health.

[25] Hargreaves T. Practice-ing behaviour change: applying social practive theory to pro-environmental behaviour change. J Consum Cult. 2011;11(79).

[26] Shove E, Trentmann F (2018). Infrastructure in practice: the dynamics of demand in networked societies.

[27] Hampton S (2018). Policy implementation as practice? Using social practice theory to examine multi-level governance efforts to decarbonise transport in the United Kingdom. Energy Res Soc Sci.

[28] Bunn C, Wyke S, Gray C, Maclean A, Hunt K (2016). ‘Coz football is what we all have’: masculinities, practice, performance and effervesce in gender-sensitized weight-loss and healthy living programme for men. Sociology Health Illness.

[29] Shaw J, Shaw S, Wherton J, Hughes G, Greenhalgh T (2017). Studying scale-up and spread as social practice: Theoretical introduction and empirical case study. J Med Internet Res.

[30] Lothian Health Board (2011). The heart manual (post MI edition).

[31] Greaves CJ, Wingham J, Deighan C (2016). Optimising self-care support for people with heart failure and their caregivers: development of the rehabilitation Enablement in chronic heart failure (REACH-HF) intervention using intervention mapping. Pilot Feasibility Stud.

[32] Frost J, Wingham J, Britten N, Greaves C, Abraham C, Warren F, Jolly K, Doherty P, Miles J, Singh S, Paul K, Taylor R, Dalal H (2019). Home-based rehabilitation for heart failure with reduced ejection fraction: process evaluation of the REACH-HF multicentre randomised controlled trial. BMJ Open.

[33] American Thoracic Society. Minnesota living with heart failure questionnaire, New York: American Thoracic Society, 2004. . http://qol.thoracic.org/sections/instruments/ko/pages/mlwhfq.html.

[34] EuroQoL G (1990). EuroQol – a new facility for the measurement of health-related quality of life. Health Policy.

[35] Zigmond AS, Snaith RP (1983). The hospital anxiety and depression scale. Acta Psychiatr Scand.

[36] Pulz C, Diniz RV, Alves AN (2008). Incremental shuttle and six-minute walking tests in the assessment of functional capacity in chronic heart failure. Can J Cardiol.

[37] Van den Berg-Emons HJ, Bussmann JB, Balk AH (2000). Validity of ambulatory accelerometry to quantify physical activity in heart failure. Scand J Rehabil Med.

[38] Oldridge N, Hofer S, McGee H (2014). The HeartQoL: part II. Validation of a new core health-related quality of life questionnaire for patients with ischemic heart disease. Eur J Prev Cardiol.

[39] Riegel B, Lee CS, Dickson VV (2009). An update on the self-care of heart failure index. J Cardiovasc Nurs.

[40] Criteria Committee of the New York Heart Association (1994). Nomenclature and criteria for diagnosis of diseases of the heart and great vessels.

[41] StataCorp (2015). Stata statistical software: release 14.

[42] QSR International Pty Ltd (2012). NVivo qualitative data analysis software; version 10.

[43] Bazeley P. Integrating analysis in mixed methods research. London; Sage. 2018.

[44] Miles M, Huberman A, Saldana J (2014). Qualitative data analysis: a methods sourcebook.

[45] Saldana J (2003). Longitudinal qualitative research: Analysing change through time.

[46] Hoffman T, Glasziou P, Boutron I, Milne R, Perera R, Moher D, Altman D, Barbur V, Macdonald H, Johnston M, Dixon-Woods M, McCulloch P, Wyatt J, Chan A, Michie S (2014). Better reporting of interventions: template for intervention description and replication (TIDieR) checklist and guide. Br Med J.

[47] Davidoff F, Dixon-Woods M, Leviton L, Michie S (2015). Demystifying theory and its use in improvement. BMJ Qual Saf.

[48] Dalal H, Doherty P, Taylor R (2015). Cardiac rehabilitation. Br Med J.

[49] Gallacher K, May C, Montori V, Mair F (2011). Understanding Patients’ Experiences of Treatment Burden in Chronic Heart Failure Using Normalization Process Theory. Ann Fam Med.

[50] Blue S, Shove E, Carmona C, Kelly M. Theories of practice and public health: understanding (un)healthy practices. Critical Public Health. 2016;26(1):36–50.

[51] Goodyear L, Barela E, Jewiss J, Usinger J (2014). Qualitative inquiry in evaluation: from theory to practice (research methods for social sciences).

[52] O’Cathain A (2018). A practical guide to using qualitative research with randomised controlled trails.

[53] Nilsen P (2015). Making sense of implementation theories, models and framework. Implementation Science.

[54] Harvey G, Kitson A (2016). PARIHS revisited: from heuristic to integrated framework for the successful implementation of knowledge into practice. Implement Sci.

[55] Haynes A, Brennan S, Redman S, Williamson A, Gallego G, Butow P, CIPHER team (2016). Figuring out fidelity: a worked example of the methods used to identify, critique and revise the essential elements of a contextualised intervention in health policy. Implement Sci.

[56] Lowery J, Hopp F, Subramania U, Witalia W, Welsh D, Larkin A, Stemmer K, Zak C, Vaitkevicius P (2012). Evaluation of a nurse practitioner disease management model for chronic heart failure: a multi-site implementation study. Congestive Heart Failure.

[57] Macarthy M, Dickinson V, Katz S, Sciacca K (2015). Process evaluation of an exercise counselling intervention using motivational interviewing. Appl Nurs Res.

